# Mitochondrial Triglyceride Dysregulation in Optic Nerves Following Indirect Traumatic Optic Neuropathy

**DOI:** 10.3390/biom12121885

**Published:** 2022-12-15

**Authors:** Muhammad Z. Chauhan, Joseph G. Chacko, Alireza Ghaffarieh, Chloe M. Moulin, Daniel Pelaez, Sami H. Uwaydat, Sanjoy K. Bhattacharya

**Affiliations:** 1 Department of Ophthalmology, Jones Eye Institute, University of Arkansas for Medical Sciences Little Rock, Little Rock, AR 72205, USA; 2 Miami Integrative Metabolomics Research Center, Bascom Palmer Eye Institute, University of Miami Miller School of Medicine, Miami, FL 33136, USA; 3 Dr. Nasser Al-Rashid Orbital Vision Research Center, Department of Ophthalmology, Bascom Palmer Eye Institute, University of Miami Miller School of Medicine, Miami, FL 33136, USA; 4 Department of Ophthalmology, Bascom Palmer Eye Institute, University of Miami Miller School of Medicine, Miami, FL 33136, USA

**Keywords:** optic nerve, traumatic optic neuropathy, mitochondrial dysregulation, lipidomics, triglycerides, animal models

## Abstract

The purpose of this work is to identify mitochondrial optic nerve (ON) lipid alterations associated with sonication-induced traumatic optic neuropathy (TON). Briefly, a mouse model of indirect TON was generated using sound energy concentrated focally at the entrance of the optic canal using a laboratory sonifier (Branson Digital Sonifier 450, Danbury, CT, USA) with a microtip probe. We performed an analysis of a previously generated dataset from high-performance liquid chromatography-electrospray tandem mass spectrometry (LC-MS/MS). We analyzed lipids from isolated mitochondria from the ON at 1 day, 7 days, and 14 days post-sonication compared to non-sonicated controls. Lipid abundance alterations in post-sonicated ON mitochondria were evaluated with 1-way ANOVA (FDR-adjusted significant *p*-value < 0.01), debiased sparse partial correlation (DSPC) network modeling, and partial least squares-discriminant analysis (PLS-DA). We find temporal alterations in triglyceride metabolism are observed in ON mitochondria of mice following sonication-induced optic neuropathy with notable depletions of TG(18:1/18:2/18:2), TG(18:1/18:1/18:1), and TG(16:0/16:0/18:1). Depletion of mitochondrial triglycerides may mediate ON damage in indirect traumatic optic neuropathy through loss energy substrates for neuronal metabolism.

## 1. Introduction

Traumatic Optic Neuropathy (TON) is a potentially devastating complication of acute injury to the optic nerve (ON) due to trauma of the head or orbit. It is characterized according to the location of the lesion (e.g., optic nerve head, intracanalicular, intracranial) or the manner of injury (i.e., direct or indirect) [1]. Direct TON occurs when the substance of the ON is impacted due to penetrating or compression injuries. Indirect TON, on the other hand, develops following blunt force head trauma when traumatic stress is transmitted to the ON via the oculofacial soft tissues or skeleton [2]. Indirect trauma is more common than direct TON and causes vision loss through the compromise of the ONs integrity through energy absorbed at impact. The exact pathophysiological and molecular mechanisms that lead to indirect TON are currently unknown. Histopathologic alterations found after TON damage include axonal degeneration and retinal ganglion cell (RGC) death [3]. A recently developed model of indirect TON using ultrasound shockwaves (sonication-induced TON) found significant elevations in tumor necrosis factor (TNF) in ONs and reactive oxygen species (ROS) in the retina following sonication-induced trauma [4]. Our previous work on whole ON lipidomics from this model found temporal alterations in sphingolipid metabolism, suggesting that lipid membrane abnormalities may be one mediator of pathology [5]. It is well established that mitochondria are not only important in the formation of ROS, but they are also a target of oxidative stress, implicating mitochondrial dysfunction early on in the disease process. In fact, another recent work found a 21% increase in RGC survival rate following sonication-induced TON compared to naïve eyes when treated with intravitreal mitochondrial-targeted therapy with elamipretide (MTP-131) and etanercept [6]. Given that lipid metabolites and their peroxidation products act as indicators of oxidative stress, in this brief report we aimed to identify localized mitochondrial ON lipid alterations following sonication-induced TON to better elucidate the mitochondrial molecular dynamics in ON disease.

## 2. Materials and Methods

### 2.1. Animals

This work utilized publicly available data. Animals utilized for the generation of this dataset were C57BL/6J mice from Jackson Laboratory (Bar Harbor, ME, USA) and were housed in a temperature-controlled environment with a 12 h light/12 h dark cycle. All mice were fed freely. Mice were two-months-old. Sonication-induced traumatic optic neuropathy was carried out as previously reported [4]. TON was induced in C57BL/6J mice using a 3 mm microtip probe in a soundproof chamber using a Branson Digital Sonifier 450 (Branson Ultrasonics, Danbury, CT, USA). Mice were sedated using vaporized isoflurane and oxygen. Supraorbital hair was removed and the microtip probe was positioned exactly above the optic nerve’s entry site into the optic canal. The left optic nerves were wounded with a 500 msec shock at a 35% amplitude producing ultrasonic waves at a frequency of 20 kHz. After sonication, mice were transferred to a fresh cage with thermal assistance until they recovered completely.

### 2.2. Mitochondrial Isolation and Transmission Electron Microscopy

Sample preparation and mitochondrial isolation for this dataset has been previously described [7]. Briefly, 1 day, 7 days, and 14 days after sonication, optic nerve samples were collected. The brains of mice were carefully removed to reveal the optic nerves and tracts underneath. Optic nerves were extracted by dissecting them from the optic nerve head to the optic chiasm. Mitochondria were isolated using the Mitochondrial Isolation Kit for Tissue (PIERCE, Rockford, IL, USA). The resulting mitochondrial pellet was maintained separately from the supernatant for cytosolic examination. The mitochondrial pellet was washed with wash buffer and put on ice until lipid extraction could be performed. Dot blot analysis targeting TOM20, a mitochondrial membrane protein, verified mitochondrial isolation.

For TEM, at either 2, 7, or 14 days-post injury optic nerves were dissected out, trimmed to include only the canalicular portion of the nerve, and fixed in cold glutaraldehyde. The samples were embedded in resin blocks and longitudinal thin sections (100 nm) were cut for copper EM grids. Images were acquired on a JEOL JEM-1400 microscope (Miami Project to Cure Paralysis core, University of Miami, FL, USA).

### 2.3. HPLC Mass Spectrometry, Lipid Identification, and Data Analysis

**Figure 1:**
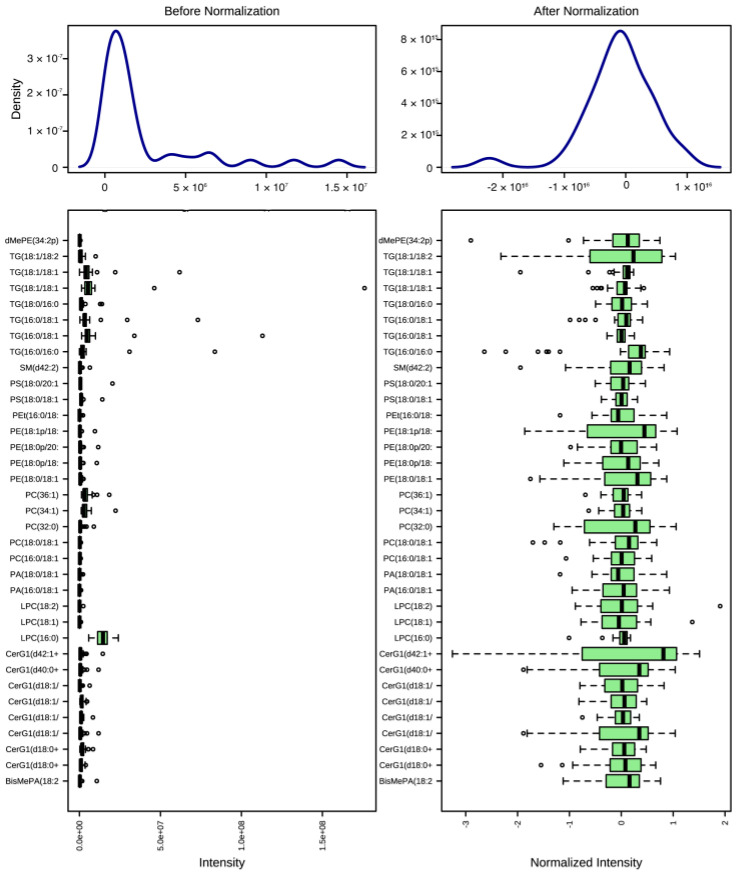
**Lipidome data before and after normalization and transformation.** Box plots and kernel density plots before and after normalization. The boxplots show 50 features due to space limitations. The density plots are based on all samples. Selected methods: Row-wise normalization: Normalization to constant sum; Data transformation: Log10 transformed; Data scaling: mean-centered.

Lipid samples were resuspended in 50 µL chloroform: methanol 2:1 (*v*/*v*) and put in an ultrasonic water bath for 20 min, followed by a 2 min vortex. 30 µL were placed into vials for four runs of 5 µL each (two positive, two negative). The lipids were examined using liquid chromatography electrospray tandem mass spectrometry (LC-MS/MS) and an orbitrap mass spectrometer (Q-Exactive, Thermo Scientific, Waltham, MA, USA). A Thermo Scientific Acclaim 120 C18 3µm column was used with LC-MS grade methanol: water 60:40 (*v*/*v*) with 10 mM ammonium acetate as solvent A and methanol chloroform 60:40 (*v*/*v*) with 10 mM ammonium acetate as solvent B. A Heated Electrospray Ionization Source (HESI) was operated at a spray voltage of 4415 V, a vaporization temperature of 275 °C, and a 15 arbitrary unit auxiliary gas flow. 150–1500 *m*/*z* was chosen as the scan range. For 13 min, the gradient flowed from 35% to 100% Solvent B, was held at 35% Solvent B for 2 min, and was eventually raised up to 100% Solvent A for 3 min and held for 2 min. Validation of the LC-MS/MS technique for mitochondrial lipid analysis was performed using external standards containing established quantities of cardiolipin. LipidSearch 4.1.3 was used to upload the raw data generated by LC-MS/MS. The settings were adjusted to an M-score of 5.0, Productsearch, precursor (5/5) ppm, 1.0 percent intensity threshold, and quantitation and TopRank filtering were enabled. Identification quality was assessed on a scale of A-D. All target classes have been chosen. Except for (CH_3_CH_2_)_3_NH^+^ and (CH_3_)_2_NH_2_, all adducts were chosen for both negative and positive modes. After identifying all peaks, samples were aligned in positive mode, negative mode, and also throughout all trial runs. Of note, ion mobility prior to the mass spectrometry was not performed thus results may improve with the use of field asymmetric ion mobility (FAIM) prior to mass spectrometry. Identification of lipids was assessed on a scale of A-C with an M-Score of 5. Any peaks that were determined to be false positives were discarded. Statistical analysis was performed using MetaboAnalyst 5.0 [8]. Prior to analyses data were filtered by interquartile range, normalized by sum, log_10_ transformed and mean-centered in order to remove skewness and nonbiological, procedure-based variation (Figure 1).

## 3. Results

**Figure 2:**
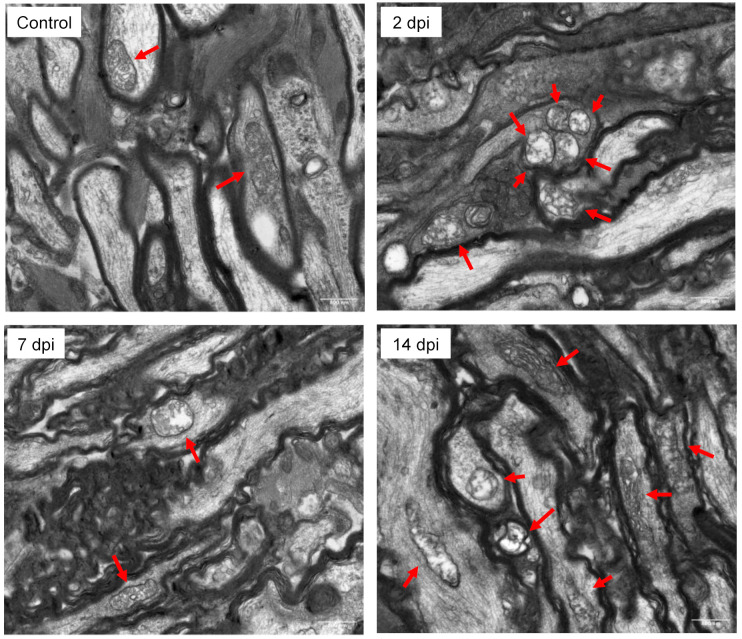
**Transmission electron microscopy (TEM) micrographs of longitudinal thin sections from mouse optic nerves.** Representative micrograph of an uninjured (control) optic nerve with normal mitochondria that exhibit dense inner matrices with intact outer membranes. At 2-, 7-, and 14-day post injury (dpi), mitochondria exhibit vesicular-swollen and swollen phenotypes. Swollen mitochondria exhibit expanded matrix space, lack of staining of the matrix, and fragmented or disorganized cristae. Red arrows point to mitochondria in the images.

We performed high-performance liquid chromatography (HPLC) tandem mass spectrometry (LC-MS/MS) of isolated mitochondrial from the ON of 8 control C57BL/6J mice and in 24 mice following sonication-induced trauma: 1 day (*n* = 8), 7 days (*n* = 8), and 14 days (*n* = 8) post-exposure. Transmission electron microcopy of mitochondria at 2-, 7-, and 14-day post injury (dpi), revealed vesicular-swollen and swollen phenotypes compared to control. Swollen mitochondria exhibit expanded matrix space, lack of staining of the matrix, and fragmented or disorganized cristae (Figure 2).

**Figure 3:**
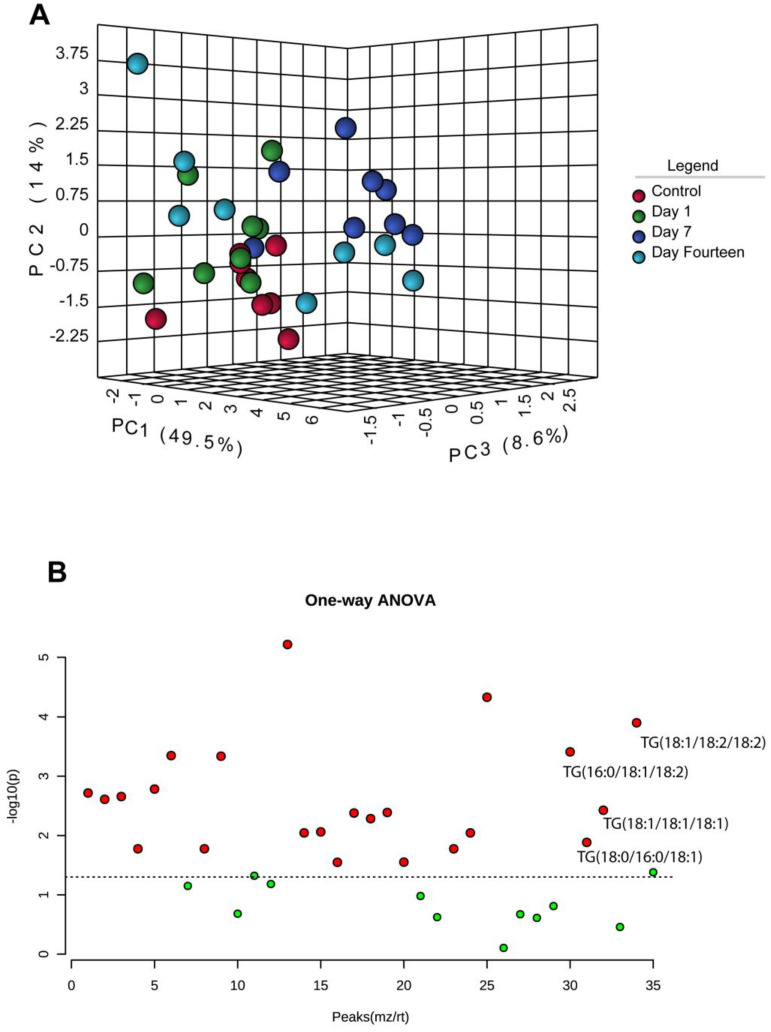
**Optic nerve mitochondrial lipidomic profiling.** (**A**) Three-dimensional unsupervised scores plot between the selected principal components (PC) across control and post-sonication groups (1-day, 7-day, and 14-day). The explained variances are shown in brackets. The first two principal components (PC1 and PC2) explaining 63.5% of the variance. (**B**) Differentially expressed TG lipids from isolated ON mitochondria compared using 1-way ANOVA analysis. Important lipid species were selected by ANOVA plot with an FDR adjusted *p*-value (0.05).

A 3D unsupervised principal component analysis (PCA) revealed clustering of samples at 14 days after sonication with the score for the first two principal components (PC1 and PC2) explaining 63.5% of the variance (Figure 3A). An FDR adjusted one-way ANOVA revealed 23 lipid species with differential abundances across control and experimental groups. We found four triglyceride (TG) species were significantly different across groups: TG(18:1/18:2/18:2), TG(16:0/18:1/18:2), TG(18:1/18:1/18:1), and TG(18:0/16:0/18:1) (*p* < 0.01) (Figure 3B).

**Figure 4:**
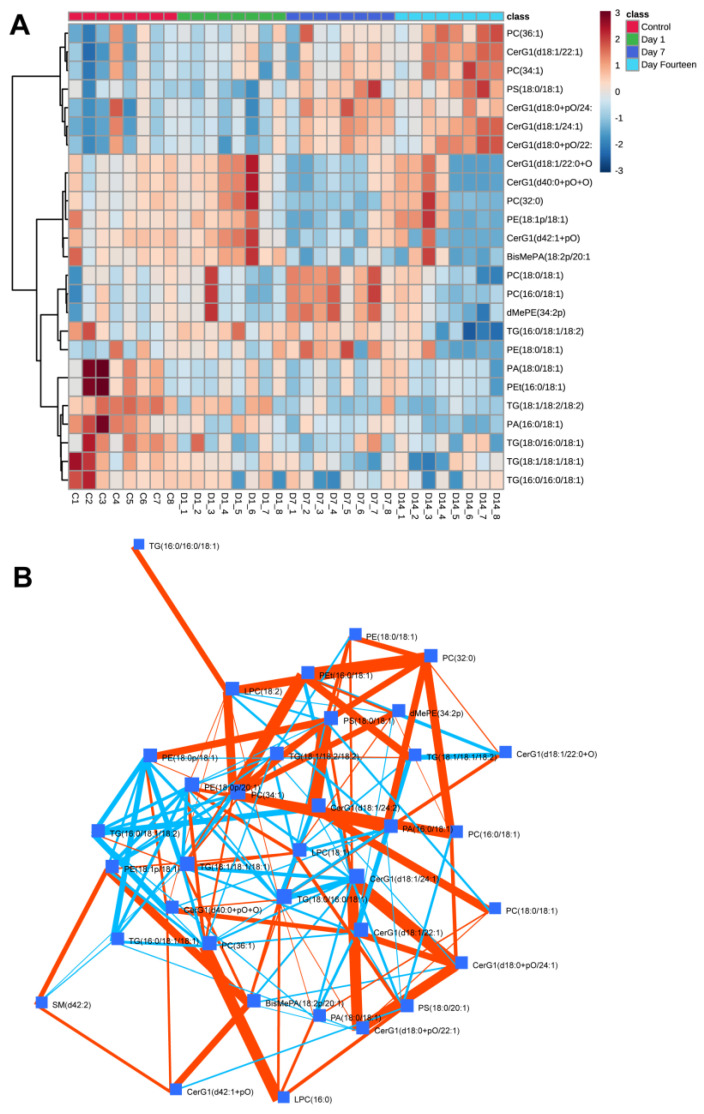
**Temporal and connectivity lipidomic patterns in ON mitochondria following sonication-induced TON.** (**A**) Heatmap of significantly different lipid species across control and post-sonication groups (1-day, 7-day and 14-day); Euclidian distance measure and Ward clustering algorithm (red denotes enrichment, blue denotes depletion). TG lipid species were found to have a stepwise decrease in abundance. (**B**) Debiased sparse partial correlation (DSPC) network modeling revealed a significant positive correlation (red) between TG lipid species, while TGs were negatively correlated (blue) with several hexosylceramide (CerG1) and phosphatidylethanolamine (PE) species.

A heat map of significantly different mitochondrial lipid species from ANOVA found a stepwise deletion of multiple lipid classes from day 1 to day 14 post-sonication (Figure 4A). All TG species were found to have a stepwise decrease in abundance. Several hexosylceramide (CerG1) lipid species showed the opposite trend. Debiased sparse partial correlation (DSPC) network modeling was utilized to uncover connectivity patterns and correlations across lipid species (Figure 4B). TG lipid species were found to be significantly positively correlated with each other (red) while negatively correlated with several CerG1 and phosphatidylethanolamine (PE) species.

**Figure 5:**
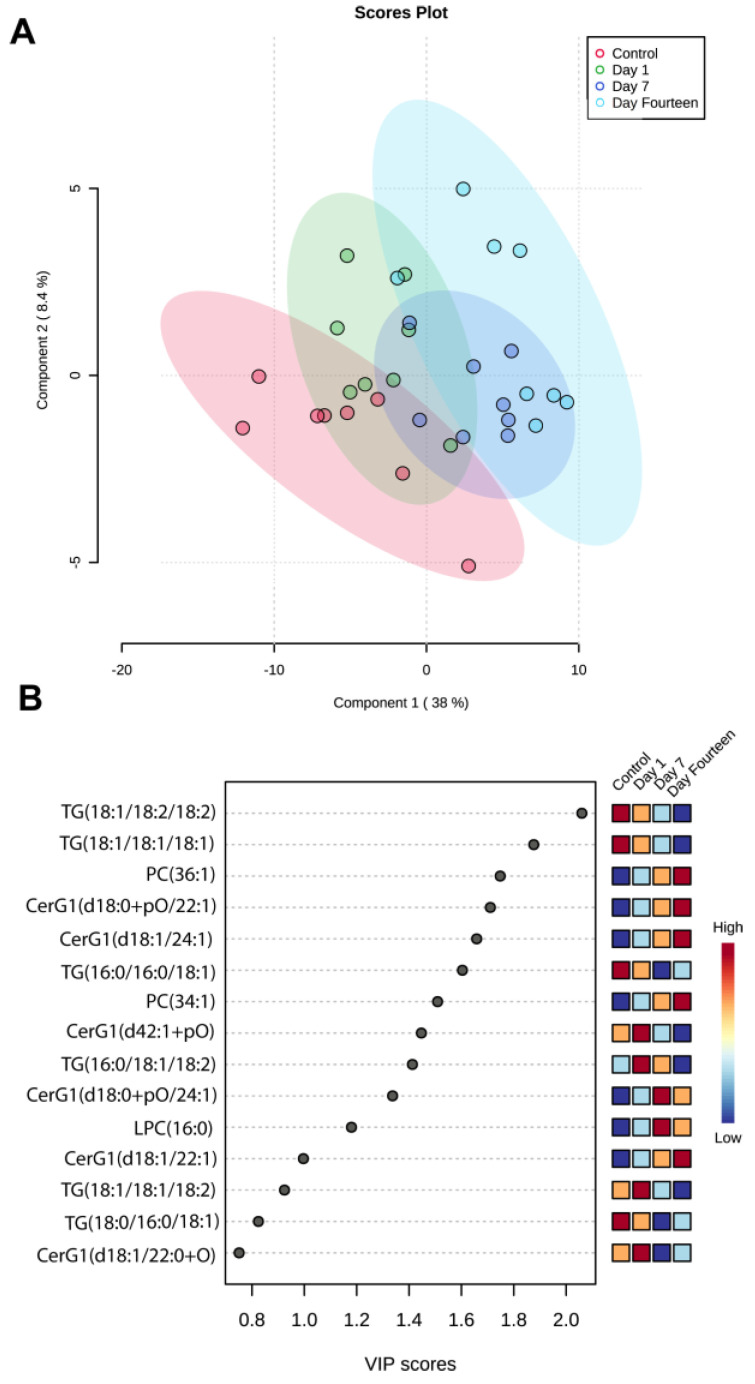
**Partial least squares-discriminant analysis (PLS-DA)**. (**A**) Partial least squares-discriminant analysis two-dimensional scores plot showed that component 1 explained 38% of the variance. Post-sonication groups were found to be more distinct from control. (**B**) Important features identified by PLS-DA. Top 15 lipid species found through variable importance in projection (VIP) analysis.

We subsequently performed a partial least squares-discriminant analysis (PLS-DA) to identify lipid species that most significantly predict control and post-sonication groups (R^2^ = 0.91601 and Q^2^ = 0.62133, accuracy: 0.59375) (Figure 5A). A 2D scores plot showed that component 1 explained 38% of the variance and revealed a difference in lipidome between control and post-sonication groups. Further examining the top 15 lipid species through variable importance in projection (VIP) analysis, it was confirmed that several TG species had a stepwise depletion from the control group to day 14 post sonication. It was found that TG(18:1/18:2/18:2) and TG(18:1/18:1/18:1) had the highest VIP scores (2.1 and 1.9, respectively), and were depleted at day 14. In addition, three CerG1 species were found to have the opposite trend and be in top 15 features from PLS-DA: CerG1(d18:0 + pO/22:1), CerG1(d18:1/24:1), and CerG1(d18:1/24:1). These CerG1 species were found to have a stepwise enrichment from control to day 14 post-sonication. Two phosphatidylcholine (PC) species (36:1 and 34:1) were also found to follow a similar pattern (Figure 5B).

## 4. Discussion

In this work, we aimed to determine the changes in the mitochondrial lipidome following sonication-induced traumatic optic neuropathy in comparison to non-sonicated controls. Our previous work examined changes in the whole optic nerve and found notable alterations in cellular sphingomyelin and hexosylceramide species. Specifically, we found that elevations in CerG1(d18:1/24:2) were linked to ON damage following indirect trauma, suggesting that pathologic lipid membrane abnormalities may contribute to etiology. We add to this work by examining organelle-specific changes in the mitochondria in the same model. We find that at 2-, 7-, and 14-day post-sonication injury, mitochondria exhibit swollen phenotypes with steady declines in mitochondrial triglyceride species, notably TG(18:1/18:2/18:2), TG(18:1/18:1/18:1), and TG(16:0/16:0/18:1).

The critical malfunctions of mitochondria in neurodegeneration are extensively recognized [9]. The mitochondria throughout the optic nerve play a protective role by providing energy to support the health and normal function of axons. Retinal ganglion cells are highly susceptible to mitochondrial dysfunction with the highest density of mitochondria found at the optic nerve head [10]. It has been shown that compared to myelinated post-laminar areas, pre-laminar and laminar regions of the optic nerve devoid of myelination have increased mitochondrial enzyme activity [11]. The high density of mitochondria in these unmyelinated areas reflects a higher energy demand than the myelinated axons. Therefore, the pre-laminar portion of the optic nerve might be considered as a susceptible metabolic chokepoint, amplifying the effects of any mitochondrial dysfunction in these areas [12]. Mitochondrial dysfunction has been linked to a number of neurodegenerative diseases, including multiple sclerosis [13], Alzheimer’s disease [14], amyotrophic lateral sclerosis (ALS) [15], and dominant optic atrophy [16]. One of the more prominent examples of mitochondrial dysfunction leading to optic nerve degeneration is Leber’s hereditary optic neuropathy (LHON), which is a genetic disorder that is caused by mitochondrial DNA mutations in the genes that encode proteins involved in the production of energy in the mitochondria [12]. Metabo-lipidomic analysis of fibroblast cell lines harboring primary LHON mutations has revealed distinct metabolomic and lipidomic signatures, with notable alterations in cellular sphingolipids and phosphatidylcholines, implicating mitochondrial dysfunction with global lipidome alterations [17]. Mitochondrial dynamics are governed in part by lipid composition, with species such as mono-, di-, and tri-glycerides, phosphatidylethanolamines, phsophatidylserines, and cholesterol needed for membrane curvature [18]. It is believed that decreases in the amount of these lipids inside the mitochondria create larger and fragmented mitochondria [19]. In fact, the administration of caprylic triglyceride, a medium-chain TG, has been shown to provide an alternate energy substrate for neuronal metabolism and improve motor impairment in the setting of ALS [20]. Additionally, the upregulation of TGs has been implicated in growth cone dynamics [21,22].

Chronic mitochondrial dysregulation has been linked to abnormalities in cellular lipid metabolism and TG dysregulation. A work by Vankoningsloo et al. (2005) explored preadipocytes response to mitochondrial dysfunction [23]. The authors found that upon administration of a mitochondrial inhibitor, there was a notable decrease in fatty acid β-oxidation and downregulation of carnitine palmitoyltransferase-1 (CPT-1), an important enzyme for mitochondrial β-oxidation, with an accumulation of cytosolic TGs. CPT-1 functions to connect carnitines to long-chain fatty acids so that they may be able to cross the inner membrane of mitochondria to be metabolized to produce energy. These findings suggest that mitochondrial damage may lead to changes in fatty acid transport into the mitochondria and loss of energy substrates. More recently, there has been a growing interest in mitochondrial fatty acid synthesis (mtFAS) with the identification of patients with neurogenerative disease harboring mutations in a mtFAS enzymes [24]. One work found mutations in *MECR* (mitochondrial trans-2-enoyl-coenzyme A-reductase), which is involved in human mtFAS, in seven individuals who presented with childhood-onset dystonia and optic atrophy [25]. Much is still to be learned on mtFAS and its role in neurodegeneration.

In our analyses, we found four mitochondrial TG lipid species that steadily declined following sonication: TG(18:1/18:2/18:2), TG(16:0/18:1/18:2), TG(18:1/18:1/18:1), and TG(18:0/16:0/18:1). The cause of mitochondrial TG loss after sonication is not clear, but may be due to loss of transport or synthesis. Our findings suggest a potential role for mitochondrial energy metabolism in the pathogenesis of indirect TON. Future research should explore the role of TGs in TON pathophysiology and the impact of TG administration on vision loss prevention. The exact cause of TG dysregulation was not explored in this work. Future studies will examine if TG loss was due to increased consumption or reduced synthesis.

The impact of indirect trauma on the retina in this model has yet to be explored. Previous works on a different model of TON found that expression levels of phospholipid species were affected by traumatic optic neuropathy and may be associated with phospholipid species-specific functions [26]. Traumatic retinopathy brought on by either direct or indirect damage to the globe is referred to as commotio retinae. It is estimated that approximately 30% of patients with eye trauma that are treated in hospitals have some form of commotio retinae. Orbital deformation from orbital trauma or blast trauma transmits shockwaves that may damage both the optic nerve and retina. An important future course would be to explore the impact of sonication on the retina at both the functional and molecular levels. Additionally, an exploration of mitochondrial changes should be examined.

## 5. Conclusions

Lipidomic analysis is a useful technique for elucidating the molecular basis of a variety of ocular disorders and for discovering new lipid signatures associated with illness. Targeted lipid study of optic neuropathy is very limited in the literature. The targeted mitochondrial lipid analysis in the present study provides valuable information not only on lipidomic results, but also on a unique strategy in metabolic functional evaluation. We found temporal alterations in triglyceride metabolism with notable depletions of TG(18:1/18:2/18:2), TG(18:1/18:1/18:1), and TG(16:0/16:0/18:1). Depletion of mitochondrial triglycerides may mediate ON damage in indirect traumatic optic neuropathy through loss energy substrates for neuronal metabolism.

## Data Availability

All data utilized in this study is available at Metabolomics Workbench [www.metabolomicsworkbench.org (Project ID: PR000905) (accessed on 1 February 2022). Metabolomics Workbench is an effort of NIH Common Fund’s Metabolomics Data Repository and Coordinating Center supported by U2C DK119886. A thorough description of the dataset and methods have been published previously in *Data-in-Brief* [7]. The sonication-induced TON model used in this work has also been detailed previously [4,5].

## References

[1] Yu-Wai-Man P. (2015). Traumatic optic neuropathy-Clinical features and management issues. Taiwan J. Ophthalmol..

[2] Sarkies N. (2004). Traumatic optic neuropathy. Eye.

[3] Singman E.L., Daphalapurkar N., White H., Nguyen T.D., Panghat L., Chang J., McCulley T. (2016). Indirect traumatic optic neuropathy. Mil. Med. Res..

[4] Tao W., Dvoriantchikova G., Tse B.C., Pappas S., Chou T.H., Tapia M., Porciatti V., Ivanov D., Tse D.T., Pelaez D. (2017). A Novel Mouse Model of Traumatic Optic Neuropathy Using External Ultrasound Energy to Achieve Focal, Indirect Optic Nerve Injury. Sci. Rep..

[5] Chauhan M.Z., Phillips P.H., Chacko J.G., Warner D.B., Pelaez D., Bhattacharya S.K. (2022). Temporal Alterations of Sphingolipids in Optic Nerves after Indirect Traumatic Optic Neuropathy. Ophthalmol. Sci..

[6] Tse B.C., Dvoriantchikova G., Tao W., Gallo R.A., Lee J.Y., Ivanov D., Tse D.T., Pelaez D. (2020). Mitochondrial targeted therapy with elamipretide (MTP-131) as an adjunct to tumor necrosis factor inhibition for traumatic optic neuropathy in the acute setting. Exp. Eye Res..

[7] Nuesi R., Gallo R.A., Meehan S.D., Nahas J.V., Dvoriantchikova G., Pelaez D., Bhattacharya S.K. (2020). Mitochondrial lipid profiling data of a traumatic optic neuropathy model. Data Brief.

[8] Pang Z., Chong J., Zhou G., de Lima Morais D.A., Chang L., Barrette M., Gauthier C., Jacques P.E., Li S., Xia J. (2021). MetaboAnalyst 5.0: Narrowing the gap between raw spectra and functional insights. Nucleic Acids Res..

[9] Raha S., Robinson B.H. (2000). Mitochondria, oxygen free radicals, disease and ageing. Trends Biochem. Sci..

[10] Muench N.A., Patel S., Maes M.E., Donahue R.J., Ikeda A., Nickells R.W. (2021). The Influence of Mitochondrial Dynamics and Function on Retinal Ganglion Cell Susceptibility in Optic Nerve Disease. Cells.

[11] Bristow E.A., Griffiths P.G., Andrews R.M., Johnson M.A., Turnbull D.M. (2002). The distribution of mitochondrial activity in relation to optic nerve structure. Arch. Ophthalmol..

[12] Yu-Wai-Man P., Griffiths P.G., Chinnery P.F. (2011). Mitochondrial optic neuropathies—Disease mechanisms and therapeutic strategies. Prog. Retin. Eye Res..

[13] Bergaglio T., Luchicchi A., Schenk G.J. (2021). Engine Failure in Axo-Myelinic Signaling: A Potential Key Player in the Pathogenesis of Multiple Sclerosis. Front. Cell. Neurosci..

[14] Sharma V.K., Singh T.G., Mehta V. (2021). Stressed mitochondria: A target to intrude Alzheimer’s disease. Mitochondrion.

[15] Ehinger J.K., Morota S., Hansson M.J., Paul G., Elmer E. (2015). Mitochondrial dysfunction in blood cells from amyotrophic lateral sclerosis patients. J. Neurol..

[16] Lenaers G., Neutzner A., Le Dantec Y., Juschke C., Xiao T., Decembrini S., Swirski S., Kieninger S., Agca C., Kim U.S. (2021). Dominant optic atrophy: Culprit mitochondria in the optic nerve. Prog. Retin. Eye Res..

[17] Chao de la Barca J.M., Simard G., Amati-Bonneau P., Safiedeen Z., Prunier-Mirebeau D., Chupin S., Gadras C., Tessier L., Gueguen N., Chevrollier A. (2016). The metabolomic signature of Leber’s hereditary optic neuropathy reveals endoplasmic reticulum stress. Brain.

[18] Furt F., Moreau P. (2009). Importance of lipid metabolism for intracellular and mitochondrial membrane fusion/fission processes. Int. J. Biochem. Cell Biol..

[19] Pollard A.K., Ortori C.A., Stöger R., Barrett D.A., Chakrabarti L. (2017). Mouse mitochondrial lipid composition is defined by age in brain and muscle. Aging.

[20] Zhao W., Varghese M., Vempati P., Dzhun A., Cheng A., Wang J., Lange D., Bilski A., Faravelli I., Pasinetti G.M. (2012). Caprylic triglyceride as a novel therapeutic approach to effectively improve the performance and attenuate the symptoms due to the motor neuron loss in ALS disease. PLoS ONE.

[21] Chauhan M.Z., Arcuri J., Park K.K., Zafar M.K., Fatmi R., Hackam A.S., Yin Y., Benowitz L., Goldberg J.L., Samarah M. (2020). Multi-Omic Analyses of Growth Cones at Different Developmental Stages Provides Insight into Pathways in Adult Neuroregeneration. IScience.

[22] Martin R.E., Bazan N.G. (1992). Changing fatty acid content of growth cone lipids prior to synaptogenesis. J. Neurochem..

[23] Vankoningsloo S., Piens M., Lecocq C., Gilson A., De Pauw A., Renard P., Demazy C., Houbion A., Raes M., Arnould T. (2005). Mitochondrial dysfunction induces triglyceride accumulation in 3T3-L1 cells: Role of fatty acid beta-oxidation and glucose. J. Lipid Res..

[24] Nowinski S.M., Van Vranken J.G., Dove K.K., Rutter J. (2018). Impact of Mitochondrial Fatty Acid Synthesis on Mitochondrial Biogenesis. Curr. Biol..

[25] Heimer G., Kerätär J.M., Riley L.G., Balasubramaniam S., Eyal E., Pietikäinen L.P., Hiltunen J.K., Marek-Yagel D., Hamada J., Gregory A. (2016). MECR Mutations Cause Childhood-Onset Dystonia and Optic Atrophy, a Mitochondrial Fatty Acid Synthesis Disorder. Am. J. Hum. Genet..

[26] Hirahara Y., Wakabayashi T., Koike T., Gamo K., Yamada H. (2020). Change in phospholipid species of retinal layer in traumatic optic neuropathy model. J. Neurosci. Res..

